# Integrated Multi-Omics Reveals the Molecular Basis Underlying Wheat Grain Development and Identifies *TaYAK1-2D* as a Positive Grain Weight Regulator

**DOI:** 10.3390/plants14243868

**Published:** 2025-12-18

**Authors:** Yazhou Xuan, Ling Zhao, Yinuo Li, Shujing Guo, Yuxue Pan, Liuge Xue, Hualiang Qiao, Wenzhao Xie, Lin Guo, Baowen Zhang, Shuzhi Zheng, Xigang Liu, Wenqiang Tang, Chunjiang Zhou, Lei Wang, Jun Ji, Junming Li, Hong Liu

**Affiliations:** 1 Ministry of Education Key Laboratory of Molecular and Cellular Biology, Hebei Collaboration Innovation Center for Cell Signaling, Hebei Key Laboratory of Molecular and Cellular Biology, College of Life Sciences, Hebei Normal University, Shijiazhuang 050024, China; 2 Institute of Millet Crops, Key Laboratory of Genetic Improvement and Utilization for Featured Coarse Cereals, Ministry of Agriculture and Rural Afairs, Hebei Academy of Agriculture and Forestry Sciences, Shijiazhuang 050035, China; 3 Center for Agricultural Resources Research, Institute of Genetics and Developmental Biology, Chinese Academy of Sciences, Shijiazhuang 050022, China

**Keywords:** bread wheat, grain weight, multi-omics, *TaYAK1-2D*, haplotype analysis

## Abstract

Grain weight, a highly heritable yield component, is a primary breeding target for enhancing wheat productivity. Unraveling the molecular dynamics underlying grain development is essential for identifying key regulators controlling this trait. In this study, we employed an integrated multi-omics approach to analyze transcriptomic and proteomic profiles in developing grains using pairwise near-isogenic lines with contrasting grain weight across four grain developmental stages. Our analysis revealed that early grain development, particularly at 7 days post-anthesis, serves as a critical window during which differential regulation of genes and proteins involved in carbohydrate biosynthesis and metabolic pathways establishes the final grain weight. By combining weighted gene co-expression network analysis (WGCNA) and K-means clustering, we identified a grain weight-associated module and pinpointed four high-confidence candidate genes. Among these, *TaYAK1-2D*, which encodes a YAK family protein kinase, was functionally validated as a positive regulator of grain weight through mutational analysis. Sequence analysis revealed two major natural haplotypes of *TaYAK1-2D*, with *TaYAK1-2D-Hap2* being significantly associated with higher grain weight across multiple environments. Our findings not only delineate a crucial metabolic window governing grain weight but also provide both a novel genetic target and a practical haplotype marker for molecular breeding aimed at yield improvement in wheat.

## 1. Introduction

Wheat (*Triticum aestivum* L.) stands as a cornerstone of global food security, with its yield potential governed by three key components: spike number per unit area, grain number per spike, and grain weight [1]. Among these, grain weight, a highly heritable trait, has long been recognized as a pivotal driver of yield improvement [2]. Therefore, uncovering the molecular regulatory networks that orchestrate grain development, along with identifying key genetic determinants controlling grain weight, is essential for advancing wheat breeding strategies. Although grain weight is closely associated with grain size [3], our understanding of its regulatory mechanisms in wheat remains fragmented compared with other cereals. In rice, for instance, a sophisticated network involving phytohormones, G protein signaling, mitogen-activated protein kinase cascades, the ubiquitin-proteasome system, and transcription factors has been well documented to regulate grain size [4,5,6,7,8,9,10]. In contrast, only a limited number of transcription factors and ubiquitin-proteasome-related genes have been functionally validated to be associated with wheat grain weight [11,12,13,14]. This knowledge gap highlights the urgent need to identify key regulators of wheat grain development and to unravel their underlying molecular networks.

Recent advances in multi-omics technologies have significantly improved our understanding of the molecular mechanisms governing grain weight. For example, transcriptomic studies have evolved from bulk tissue analyses to spatially and temporally resolved profiling. Xiang et al. (2019) compared transcriptomic profiles between hexaploid wheat and its diploid ancestors and identified many endosperm-specific genes associated with carbohydrate metabolism and starch biosynthesis [15]. Their study provided important insights into the molecular basis controlling grain filling and weight determination. In a more comprehensive effort, Zhi et al. (2023) constructed a spatiotemporal transcriptomic atlas spanning the entire grain developmental cycle [16], which revealed thousands of expressed genes and IncRNAs, and delineated key developmental transitions. More recently, Li et al. (2025) employed spatial transcriptomics in early wheat grain development and identified *TaABI3-B1* as a critical transcription factor coordinating embryogenesis and grain size [17]. Complementing these findings, Guo et al. (2025) generated a high-resolution temporal atlas capturing dynamic transcriptomic programs in wheat embryo and endosperm tissues [18], outlining major transcriptional features, revealing key regulatory modules, and identifying groups of co-expressed genes that drive specific developmental processes. These studies illustrate a clear progression from bulk tissue analysis toward spatially and temporally resolved profiling, establishing a foundational framework for understanding grain development in cereal crops.

Although significant findings were obtained through transcriptome analysis, transcriptomic-level insights alone are insufficient to fully capture the functional state of developing grains [19]. Proteomic analyses have revealed that protein abundance and translational activity undergo pronounced temporal and spatial shifts during grain development [20]. Zhang et al. (2021) conducted a comprehensive proteomic survey across multiple grain tissues and stages [21], showing that early-stage proteins are enriched in cell growth and division processes, whereas late-stage proteins are associated with stress responses. Similarly, Guo et al. (2023) constructed a translational landscape of developing grains, demonstrating that highly translated genes transition from cell division and sugar transport in early stages to storage protein and starch biosynthesis in later phases [22]. These studies highlight the dynamic and complex nature of protein-level regulation, a dimension that remains underexplored relative to transcriptomics and presents challenges in reconstructing a coherent regulatory network from multi-omics data.

Integrated multi-omics strategies have thus emerged as a powerful paradigm for dissecting complex traits in cereal crops, enabling multidimensional analysis of grain development [23]. In maize, the combination of spatial transcriptomics and histology resolved cellular heterogeneity during grain filling and identified *ZmSUT1* and *ZmSUT7*, two sucrose transporters specifically expressed in the basal endosperm transfer layer, as important regulators of grain development [24]. In rice, integrated morphological-cytological and spatiotemporal transcriptomic analyses revealed that prolonged endosperm cell division and increased cell volumes underlie grain size variation. Critical to this process is the enhanced expression of basic *Leucine Zipper* (*bZIP*) and *Nuclear Factor Y Subunit C* (*NF-YC*) transcription factors during a 5–10 day post-pollination window, which serves as a decisive developmental phase for grain size determination [25]. In wheat, coordinated transcriptomic and epigenomic profiling illustrated that chromatin accessibility and histone modifications collectively regulate starch and seed storage protein synthesis, leading to the identification and validation of *Abscisic acid insensitive 3-A1* (*TaABI3-A1*) as a grain weight regulator [26]. These examples illustrate how multi-omics integration can uncover both conserved and species-specific regulatory modules, offering valuable targets for genetic improvement of cereal crops.

Based on this, this study employed an integrated RNA sequencing (RNA-seq) and tandem mass tag (TMT/iTRAQ)-based quantitative proteomics approach to explore the molecular basis of grain weight variation in wheat. We systematically analyzed the transcriptome and proteome dynamics across four key grain development stages using paired near-isogenic lines differing in grain weight. Through integrated analyses including weighted gene co-expression network analysis (WGCNA), K-means clustering, and screening of an ethyl methyl sulfonate (EMS) mutant library, we identified a core regulatory factor, TaYAK1-2D, that governs wheat grain weight. In summary, our study establishes a comprehensive framework that integrates dynamic multi-omics data with WGCNA, functional validation, and population association analysis. This framework is designed to identify key regulatory genes during wheat grain development and to evaluate the breeding potential of favorable allelic variants.

## 2. Results

### 2.1. The Near-Isogenic Lines NIL1 and NIL2 Exhibited Significant Differences in Grain Weight

**Figure 1:**
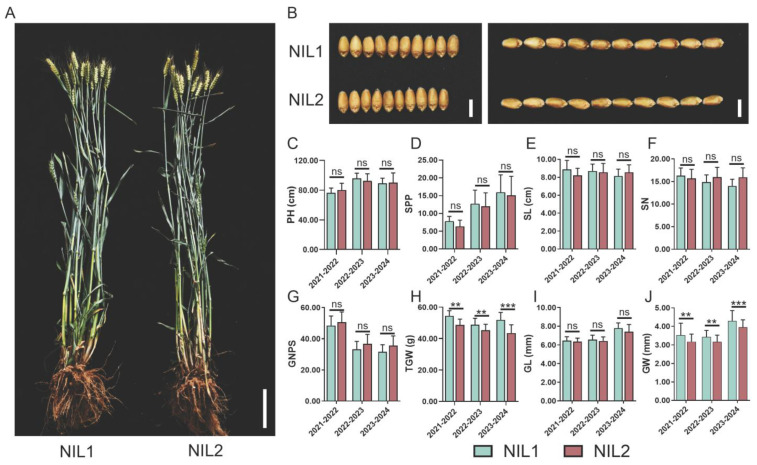
Phenotypic characterization of near-isogenic lines NIL1 and NIL2. (**A**) Representative field-grown plants of NIL1 (large grain) and NIL2 (small grain) at maturity. Scale bar, 10 cm. (**B**) Comparison of grain width and length between NIL1 and NIL2. Scale bar, 0.5 cm. (**C**–**J**) Quantitative analysis of agronomic traits: plant height (PH, (**C**)), spikes per plant (SPP, (**D**)), spike length (SL, (**E**)), spikelet number (SN, (**F**)), grain number per spike (GNPS, (**G**)), thousand-grain weight (TGW, (**H**)), grain length (GL, (**I**)), and grain width (GW, (**J**)). The *x*-axis shows the year of data collection. All values are presented as mean ± SD. Statistically significant differences are indicated by asterisks (**, *p* < 0.01; ***, *p* < 0.001, one-way ANOVA) or ns (not significant).

A pair of NILs with contrasting grain weight was selected from a BC_3_F_3_ population: NIL1 carrying large-grain alleles and NIL2 harboring small-grain alleles (Figure 1A,B). Evaluation of major agronomic traits over three consecutive growing seasons revealed no significant differences in plant height, spikes per plant, spike length, spikelet number per spike, or grain number per spike (Figure 1C–G). However, consistent and significant differences were observed in grain width (GW) and thousand-grain weight (TGW), with NIL1 exhibiting 3.60–4.51% wider GW and 8.08–12.52% higher TGW compared with NIL2 (Figure 1H–J). To investigate the molecular basis of these grain weight variations, we performed RNA-seq on developing grains from both NILs.

### 2.2. Transcriptomic Profiling Reveals Stage-Specific Expression Patterns

**Figure 2:**
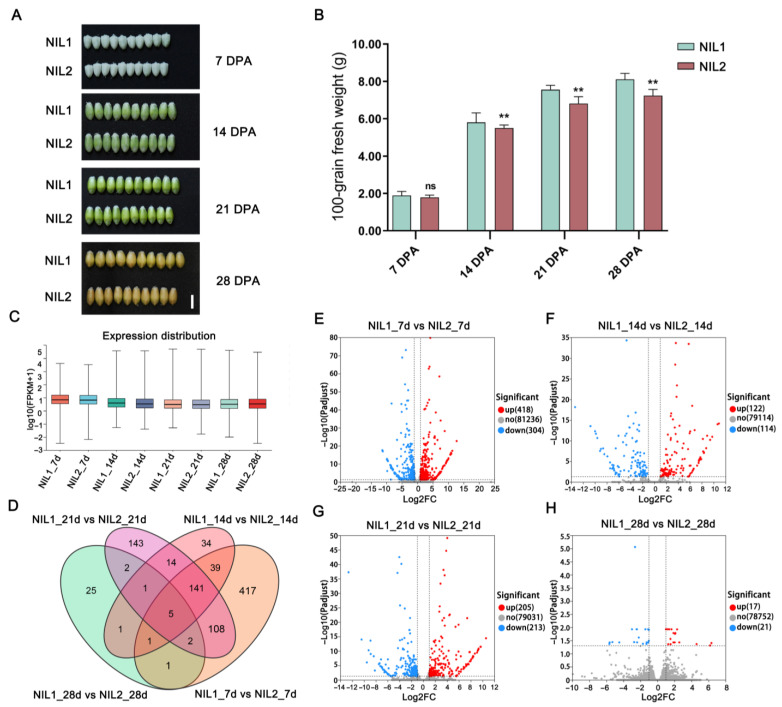
Differences in grain morphology and gene expression between NIL1 and NIL2 across different grain developmental stages. (**A**) Grain morphology of NIL1 and NIL2 at 7, 14, 21, and 28 DPA. Scale bar, 0.5 cm. (**B**) Fresh weights of NIL1 and NIL2 grains across developmental stages. Values are presented as mean ± SD, statistically significant differences are indicated by asterisks (**, *p* < 0.01, one-way ANOVA) or ns (not significant). (**C**) Global gene expression levels during grain development. (**D**) Venn diagram illustrating overlaps among DEGs at different grain developmental stages. (**E**–**H**) Volcano plots of DEGs at 7 (**E**), 14 (**F**), 21 (**G**), and 28 (**H**) DPA.

Comparative transcriptomic analysis was conducted across multiple grain developmental stages in NIL1 and NIL2. While morphological differences in grain width were not apparent at 14 days post-anthesis (DPA), fresh weight already exhibited notable variation between the two NILs at this stage (Figure 2A,B). Transcriptomic analysis showed that global gene expression peaked at 7 DPA relative to later developmental stages (Figure 2C), suggesting the importance of early grain development. Differential expression analysis between the NILs identified 714 differentially expressed genes (DEGs) at 7 DPA (410 upregulated and 304 downregulated), 236 DEGs at 14 DPA (122 upregulated and 114 downregulated), 418 DEGs at 21 DPA (205 upregulated and 213 downregulated), and only 38 DEGs at 28 DPA (17 upregulated and 21 downregulated) (Figure 2D–H). The substantially higher number of DEGs at 7 DPA suggests this stage may be pivotal in establishing the observed grain weight differences.

### 2.3. Early Grain Development Stage (7 DPA) Is Critical for Grain Weight Determination

**Figure 3:**
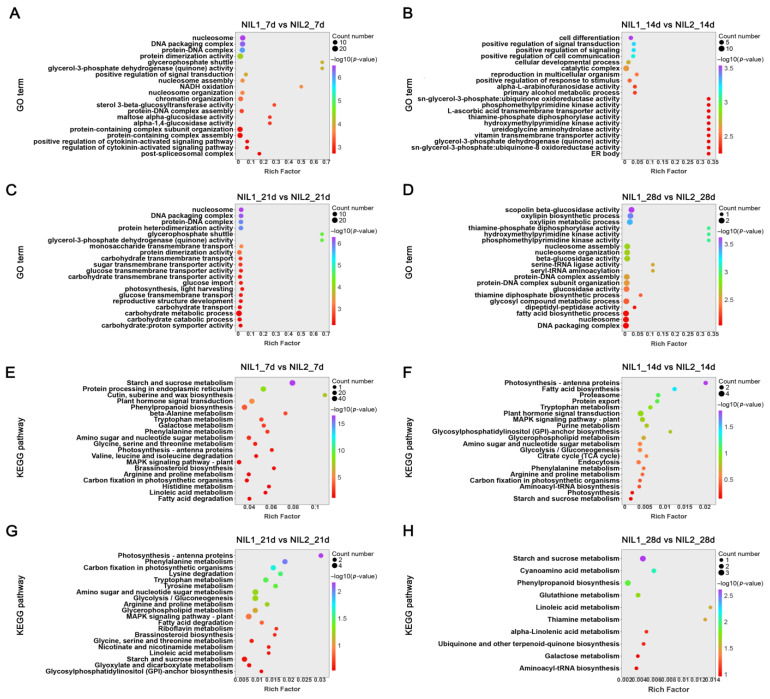
Functional enrichment analysis of DEGs during grain development between NIL1 and NIL2. (**A**–**D**) GO enrichment analysis of DEGs at 7 (**A**), 14 (**B**), 21 (**C**), and 28 (**D**) DPA. (**E**–**H**) KEGG pathway enrichment analysis of DEGs at 7 (**E**), 14 (**F**), 21 (**G**), and 28 (**H**) DPA.

Gene Ontology (GO) and Kyoto Encyclopedia of Genes and Genomes (KEGG) enrichment analyses of stage-specific DEGs revealed dynamic metabolic reprogramming during grain development. At 7 DPA, significant GO terms encompassed maltose-glucosidase activity and cytokinin-related pathways (Figure 3A), whereas processes related to cell differentiation, development, and communication became predominant at 14 DPA (Figure 3B). By 21 DPA, highlighted pathways included the glycerol-3-phosphate shuttle, monosaccharide transmembrane transport, and carbohydrate metabolism (Figure 3C), while protein biosynthesis-associated processes were prominently enriched at 28 DPA (Figure 3D). KEGG analysis further demonstrated significant enrichment in starch and sucrose metabolism, along with plant hormone signal transduction at 7 DPA (Figure 3E). This pattern shifted at 14 DPA to fatty acid biosynthesis and plant hormone signal transduction pathways (Figure 3F). At 21 DPA, carbon fixation in photosynthesis and glycolysis/gluconeogenesis pathways was significantly enriched (Figure 3G), while 28 DPA showed recurrent enrichment in starch and sucrose metabolism, accompanied by multiple amino acid metabolism pathways (Figure 3H).

Comparative analysis highlighted fundamental differences between early (7–14 DPA) and late (21–28 DPA) developmental phases. The early stages exhibited distinct variations in carbohydrate metabolism, plant hormone signaling, cell differentiation, and stress responses (Figure 3E,F). In contrast, differences in later stages were primarily associated with carbohydrate metabolism pathways (Figure 3G,H). Notably, differential regulation of carbohydrate metabolism, a process directly determining grain weight, was established as early as 7 DPA between the two NILs. Based on the significant metabolic pathway variations observed at 7 and 14 DPA during grain development, we extended our investigation to proteomic profiling to identify the protein-level changes underlying these metabolic pathway differences.

### 2.4. Grain Weight Differences Originate from Altered Carbohydrate Metabolism at 7 DPA

**Figure 4:**
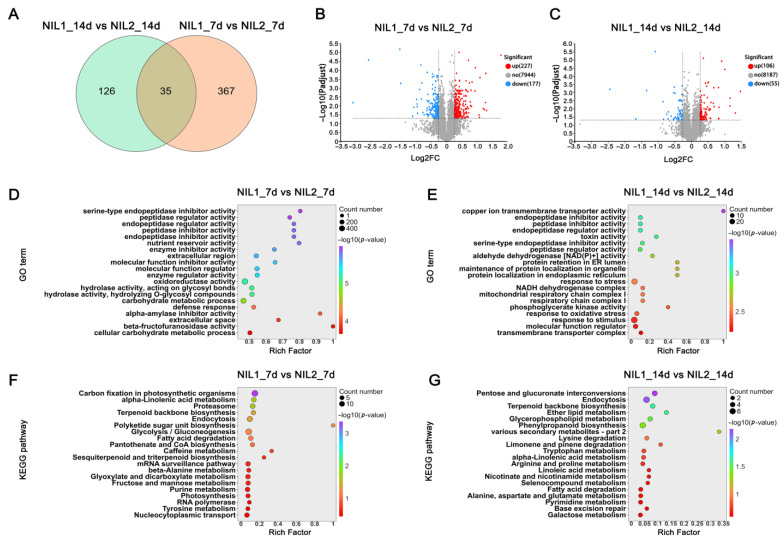
Proteomic profiling of developing grains in NIL1 and NIL2 at 7 and 14 DPA. (**A**) Venn diagram showing overlap of DEPs in grains at 7 and 14 DPA. (**B**,**C**) Volcano plots of DEPs at 7 (**B**) and 14 (**C**) DPA. (**D**,**E**) GO enrichment analysis of DEPs at 7 (**D**) and 14 (**E**) DPA. (**F**,**G**) KEGG pathway enrichment analysis of DEPs at 7 (**F**) and 14 (**G**) DPA.

Proteomic profiling identified 404 differentially expressed proteins (DEPs) at 7 DPA (227 upregulated and 177 downregulated; Figure 4A,B), compared to 162 DEPs at 14 DPA (106 upregulated and 56 downregulated; Figure 4C). GO enrichment analysis revealed distinct stage-specific patterns: at 7 DPA, DEPs were significantly enriched in carbohydrate metabolism and protein–DNA complex assembly (Figure 4D), whereas at 14 DPA, they were primarily associated with stress and external-stimulus responses (Figure 4E). KEGG pathway analysis further showed significant enrichment in carbon fixation, glycolysis, and gluconeogenesis pathways at 7 DPA (Figure 4F), whereas the endocytosis and secondary metabolite biosynthesis pathways were enriched at 14 DPA (Figure 4G).

The integration of transcriptomic DEGs and proteomic DEPs, along with their associated GO and KEGG annotations, reinforced these findings. Analysis of overlapping genes between DEGs and DEPs further revealed that DEPs at 7 DPA were primarily involved in energy metabolism processes, including carbohydrate metabolism and photosynthesis, whereas those at 14 DPA were predominantly related to stress response and secondary metabolism. More importantly, combined transcriptomic and proteomic data highlighted substantial disparities in carbohydrate metabolism at 7 DPA between NIL1 and NIL2. Expression profiling of carbohydrate metabolism-related genes showed their distinct stage-specific patterns, with most DEGs at 7 DPA showing inconsistent expression patterns at later developmental stages (Figure S1A).

To functionally validate these findings, we selected three DEGs from 7 DPA: *TraesCS4A02G116100* (xyloglucan 6-xylosytransferase), *TraesCS6B02G187500* (phosphoglycerate kinase), and *TraesCS7B02G130200* (uncharacterized protein) for further investigation using an EMS mutant library of the wheat cultivar Kenong 9204 (KN9204) [27]. Mutants for each of these genes consistently exhibited a significant reduction in TGW (Figure S1B), providing direct genetic evidence that variation in carbohydrate metabolism at 7 DPA plays a determining role in establishing final grain weight.

### 2.5. Identification of a Grain Weight-Associated Gene Cluster via K-Means Clustering

**Figure 5:**
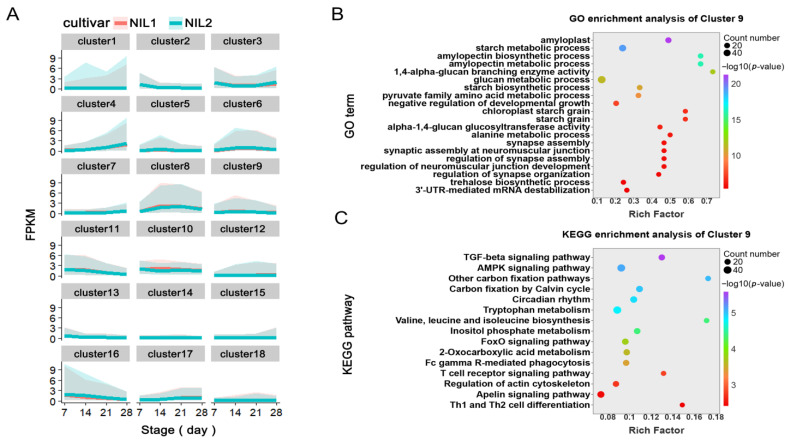
Temporal expression patterns of DEGs during grain development revealed by K-means clustering. (**A**) The expression profiles of 18 clusters identified by K-means analysis of all DEGs across four developmental stages. (**B**,**C**) Functional enrichment analysis of Cluster 9: GO terms and KEGG pathways.

To further elucidate the molecular basis of grain weight variation between NIL1 and NIL2, we performed unsupervised K-means clustering on transcriptomic data to characterize dynamic gene expression patterns during grain development. All DEGs across the four grain developmental stages were classified into 18 distinct clusters based on their expression patterns (Figure 5A), with cluster centroids, correlations, and detailed expression patterns provided in Figure S2 and Figure 5A, respectively.

The clustering revealed distinct temporal expression trajectories: genes in Clusters 1, 4, 7, and 15 maintained continuous upregulation throughout grain development, whereas those in Clusters 2, 10, 11, 13, and 14 showed progressive downregulation. Clusters 12, 17, and 18 exhibited elevated expression from 7 to 21 DPA, followed by a decline toward 28 DPA, and genes in Clusters 5, 6, 8, and 9 displayed specific peaks during 14–21 DPA.

Functional enrichment analyses of each cluster (Tables S2 and S3) identified Cluster 9 as particularly noteworthy, with significant enrichment in starch biosynthesis and carbon metabolism pathways (Figure 5B,C). This functional profile suggests that Cluster 9 genes play pivotal roles in regulating starch accumulation and carbon partitioning during grain development, positioning them as key candidates for mediating the observed differences in grain weight between NIL1 and NIL2.

### 2.6. Integrated Multi-Omics Analysis Identified Candidate Regulators of Grain Weight

**Figure 6:**
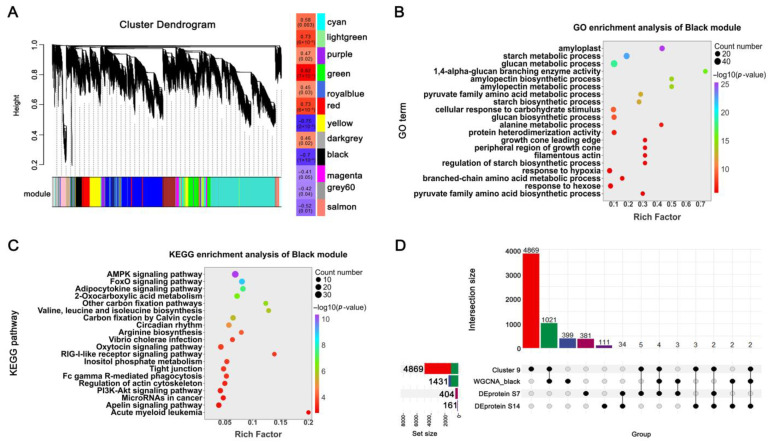
Integration of multi-omics data identifies key regulatory modules and candidate genes for grain weight control. (**A**) WGCNA module identification and correlation analysis with grain weight. (**B**,**C**) Functional enrichment analysis of the Black module in the WGCNA: GO terms and KEGG pathways. (**D**) Integrated analysis of candidate genes derived from K-means Cluster 9 (starch synthesis-related), WGCNA Black module (grain weight-associated), and DEPs.

To further identify gene networks underlying trait variation, we conducted WGCNA on the transcriptome data (Figure 6A). Genes were clustered into 12 distinct co-expression modules, each representing a unique expression trajectory and labeled with a characteristic color. Module–trait correlation analysis revealed that the Green, Red, Yellow, and Black modules showed significant associations with grain weight. Functional enrichment analyses of these modules (Tables S4 and S5) demonstrated that the Black module was particularly enriched in starch biosynthesis, carbohydrate metabolism, and energy regulation pathways (Figure 6B,C), highlighting its central role in grain weight determination.

To prioritize high-confidence regulatory candidates, we integrated multiple datasets by intersecting genes from the starch synthesis-related Cluster 9, the grain weight-associated Black module, and DEPs identified at both 7 and 14 DPA. This integrative approach yielded four consensus candidate genes consistently present across all datasets, suggesting their fundamental roles in grain weight regulation (Table 1). Among these, *TraesCS2D02G290800*, encoding Starch Branching Enzyme IIa (TaSBEIIa), has previously been established as a key enzyme in amylopectin biosynthesis [28], thereby validating the reliability of our multi-omics integration strategy. Furthermore, expression analysis indicated that *TraesCS2D02G433100* was differentially expressed between the NILs at both 7 and 14 DPA (Figure S3B), implicating it as a strong candidate for future functional studies.

### 2.7. Functional Characterization of TaYAK1-2D as a Positive Regulator of Grain Weight

**Figure 7:**
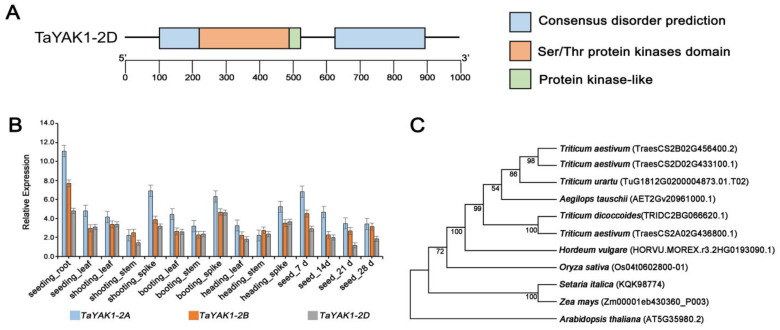
Analysis of *TaYAK1-2D* gene structure and expression patterns. (**A**) Schematic representation of gene structure and conserved protein domains. Domain annotations are shown on the right. (**B**) Expression profiles of *TaYAK1-2D* and its homologs across various tissues and developmental stages in KN9204, including roots, stems, leaves, and developing grains. (**C**) Phylogenetic analysis of *TaYAK1-2D* and its homologous proteins from representative plant species.

Sequence alignment identified *TaYAK1-2D* (*TraesCS2D02G433100*) as a member of the dual specificity YAK1-like protein kinase family (Figure S4 and Figure 7A). Phylogenetic analysis revealed high sequence conservation of TaYAK1 across monocot species, including wheat relatives, maize, and rice, suggesting evolutionary maintenance of core biological functions (Figure 7C). Tissue-specific expression profiling indicated that *TaYAK1-2D* exhibits patterns similar to its homologs *TaYAK1-2A* and *TaYAK1-2B*, with predominant expression in developing grains, spikes, and roots (Figure 7B).

**Figure 8:**
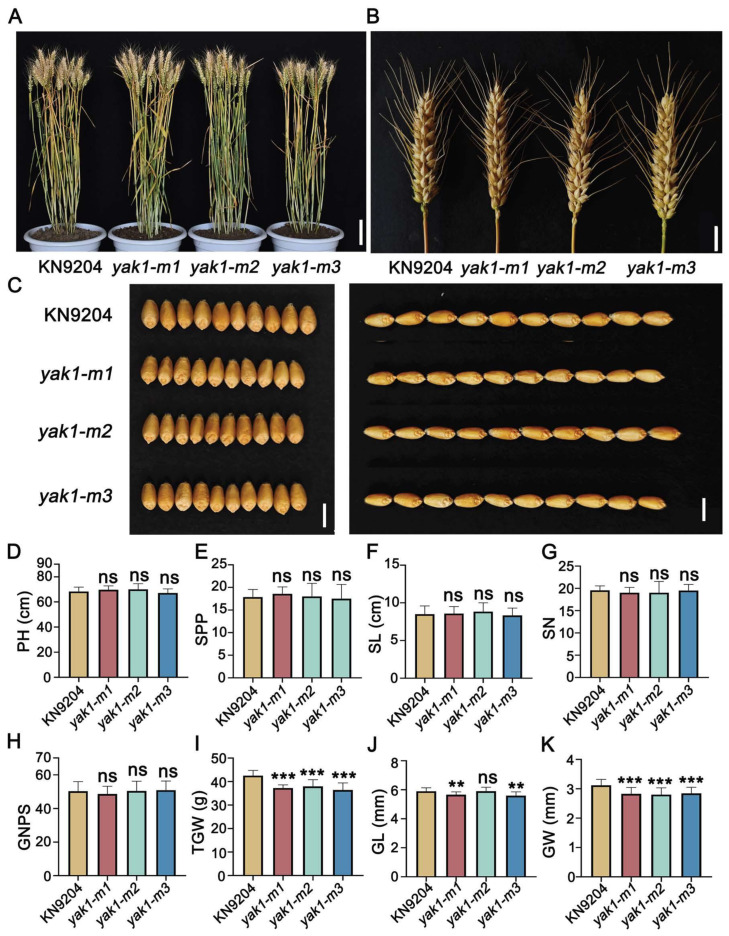
Phenotypic characterization of *yak1* EMS mutants. (**A**) Representative field-grown plants of wild type (WT) and *yak1* mutants at maturity. *yak1-m1*, *yak1-m2,* and *yak1-m3* are independent allelic mutants. Scale bar, 10 cm. (**B**) Spikes morphology of WT and *yak1* mutants. Scale bar, 2 cm. (**C**) Grain morphology comparison. Scale bar, 0.5 cm. (**D**–**K**) Quantitative analysis of agronomic traits: plant height (PH, (**D**)), spikes per plant (SPP, (**E**)), spike length (SL, (**F**)), spikelet number (SN, (**G**)), grain number per spike (GNPS, (**H**)), thousand-grain weight (TGW, (**I**)), grain length (GL, (**J**)), and grain width (GW, (**K**)). Data are presented as mean ± SD. Statistically significant differences are indicated by asterisks (**, *p* < 0.01; ***, *p* < 0.001, one-way ANOVA) or ns (not significant).

We subsequently identified and characterized loss-of-function mutants for each of the three homologs from a gene-indexed EMS mutant library of KN9204. Sequencing confirmed single-nucleotide substitutions introducing premature stop codons in all three mutants, resulting in truncated protein products (Table S6 and Figure S5). Field-grown mutants of *TaYAK1-2A*, *TaYAK1*-*2B*, and *TaYAK1-2D* showed no discernible differences from wild-type plants in major agronomic traits, including plant height, spikes per plant, spike length, and spikelet number (Figure 8A,B,D–K). However, all three mutant lines exhibited significant reductions in TGW (12.5%, 11.7%, and 15.3%, respectively), accompanied by significant decreases in both grain length and width (Figure 8I–K). These results demonstrated that disruption of *TaYAK1-2D* function specifically impairs grain weight and grain dimensions without affecting other major agronomic traits, confirming its role as a positive regulator of grain weight in wheat.

### 2.8. TaYAK1-2D Haplotype Significantly Associated with Grain Weight

**Figure 9:**
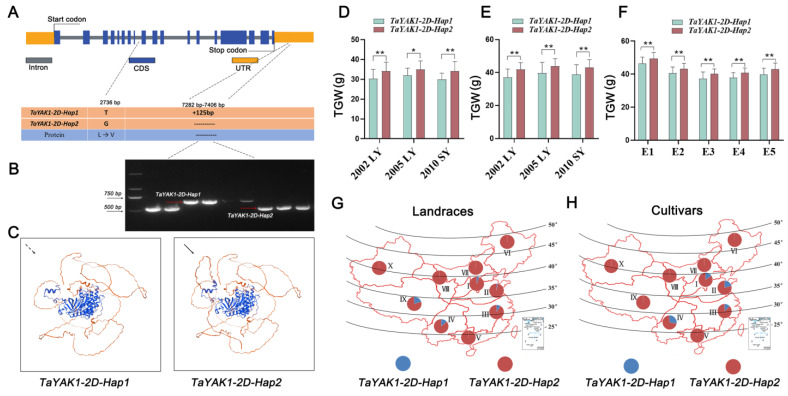
Haplotype analysis of *TaYAK1-2D* and its association with thousand-grain weight in wheat. (**A**) Gene structure of *TaYAK1-2D*, showing the SNP (2736 bp) and 125 bp InDel (7282–7406 bp) that define the two haplotypes *Hap1* and *Hap2*. (**B**) Gel electrophoresis of the InDel marker distinguishing the two haplotypes. (**C**) Homology models comparing the three-dimensional protein structures of Hap1 and Hap2. (**D**,**E**) Association analysis of *TaYAK1-2D* haplotypes with TGW in 157 landraces of the Chinese mini-core collection (MCC) and 86 modern cultivars (MC) grown in three environments: 2002 LY (Luoyang), 2005 LY, and 2010 SY (Shunyi). (**F**) Association of *TaYAK1-2D* haplotypes with TGW in 132 wheat accessions from the Yellow and Huai River valley grown in five environments (E1–E5): E1 (2015LC, Luancheng), E2 (2016LC, Luancheng), E3 (2017LC, Luancheng), E4 (17AY, Anyang), and E5 (17BJ, Beijing). Data are presented as mean ± SD. Statistically significant differences are indicated by asterisks (*, *p* < 0.05; **, *p* < 0.01, one-way ANOVA). (**G**,**H**) Geographical distribution of *TaYAK1-2D* haplotypes in landraces (**G**) and modern cultivars (**H**) across ten major Chinese wheat zones: I, northern winter wheat region; II, Yellow and Huai River valley winter wheat region; III, low and middle Yangtze River valley winter wheat region; IV, southwestern winter wheat region; V, southern winter wheat region; VI, northeastern spring wheat region; VII, northern spring wheat region; VIII, northwestern spring wheat region; IX, Qinghai–Tibet spring–winter wheat region; and X, Xinjiang winter–spring wheat region.

To explore the natural variation of *TaYAK1-2D*, we analyzed the nucleotide diversity across the genomic region (spanning 2 kb upstream of the start codon to 2 kb downstream of the stop codon) in 43 wheat accessions (Table S7). Our analysis identified a nonsynonymous single-nucleotide polymorphism (SNP) in the coding region, resulting in a leucine (Leu) to valine (Val) substitution within the conserved protein kinase domain (Figure 9C). Additionally, a 125 bp insertion/deletion (InDel) polymorphism was detected in the 3′ untranslated region (3′ UTR). These variations defined two haplotypes: *TaYAK1-2D-Hap1* and *TaYAK1-2D-Hap2* (Figure 9A).

To effectively distinguish these haplotypes, a PCR-based InDel marker (*InDel125*) was developed and validated (Figure 9B). Additionally, a KASP marker was designed based on *TaYAK1-2D* SNP loci. Genotyping with both markers, using the MCC and 132 wheat accessions, yielded fully concordant results (Figure S6, Tables S8 and S9), confirming tight linkage between the SNP and InDel loci. Subsequent association analysis revealed that *TaYAK1-2D* haplotypes are significantly correlated with TGW across multiple environments (Tables S10–S12; Figure 9D–F). Cultivars carrying *TaYAK1-2D-Hap2* consistently exhibited higher TGW compared to those with *TaYAK1-2D-Hap1*, indicating that *TaYAK1-2D-Hap*2 is the favorable haplotype. Geographical distribution analysis showed that *TaYAK1-2D-Hap1* was found only sporadically in certain wheat-growing regions and at low frequency in modern cultivars, while *TaYAK1-2D-Hap*2 predominates in both landrace and modern cultivars. These findings indicate that *TaYAK1-2D-Hap*2 has been strongly favored during wheat breeding, leading to the progressive elimination of the less advantageous *TaYAK1-2D-Hap1* haplotype.

## 3. Discussion

Grain weight is a vital determinant of yield potential and a primary breeding target in wheat [29]. Elucidating the molecular mechanisms that govern grain development is essential for identifying key regulatory genes to drive genetic improvement in agronomic yield. While multi-omics approaches have substantially accelerated the dissection of this complex trait in cereals, the regulatory networks controlling wheat grain development remain poorly characterized. In this study, we applied an integrated multi-omics analysis of NILs with contrasting grain weight, which revealed two key advances: the identification of the early grain developmental phase as a critical metabolic window determining final grain weight and the discovery of *TaYAK1-2D* as a novel positive regulator of grain weight with breeding relevance.

### 3.1. Early Metabolic Programming Determines Grain Weight Potential

Our integrated transcriptomic and proteomic analyses consistently identified 7 DPA as a decisive developmental window, with carbohydrate biosynthesis and metabolism pathways showing significant enrichment in both DEGs and DAPs (Figure 3A,E and Figure 4D,F). This developmental stage coincides with the transition from cell division to nutrient accumulation in the endosperm [30], suggesting that enhanced metabolic capacity at this early stage establishes the foundation for subsequent grain filling. The superior performance of the high-grain-weight NIL appears to stem from its enhanced capacity for sucrose and starch synthesis specifically at 7 DPA, aligning with prior evidence that early carbohydrate availability critically influences final grain weight [31,32]. Together, these findings underscore the pivotal role of early metabolic programming in determining final grain weight.

Understanding this early metabolic regulation points to the importance of key factors operating within the first week after anthesis, including transcription factors, phytohormones, and signaling molecules [17,33]. These components likely improve the efficiency of sucrose-to-starch conversion, thereby accelerating grain filling. For instance, the maize transcription factor ZmABI19 promotes grain filling by directly activating key genes like *Opaque2*, thereby coordinating starch and sucrose metabolism [34]. Similarly, gibberellin application in rice upregulates α-amylase genes (e.g., *AMY2A* and *AMY1.4*), improving both mesocotyl elongation and grain-filling efficiency [35]. Other hormones, including ethylene and abscisic acid, further modulate grain development through the regulation of starch degradation and sucrose transport genes [36]. Targeting these early regulatory nodes through gene-editing technologies, combined with marker-assisted selection strategies, could enable precise dissection of grain development mechanisms and accelerate the translation of molecular insights into yield improvement in wheat breeding.

### 3.2. Integrative Network Analysis Prioritizes High-Confidence Regulators

To further dissect the regulatory network underlying grain weight, we integrated WGCNA and K-means clustering. This combined strategy, initially proposed by Botía et al. (2017) [37], improves the identification of co-expression network structures and enhances biological interpretability and has been adopted in numerous studies. For instance, Lv et al. (2020) applied WGCNA and K-means to partition drought-responsive genes in wheat into 29 modules [38], revealing that Dark Turquoise, Yellow, and Brown modules were strongly correlated with drought resistance. They further identified 12 hub genes enriched in the drought response network. In our study, K-means clustering identified Cluster 9 as significantly enriched in starch biosynthesis and carbon metabolism pathways (Figure 5B,C). WGCNA further pinpointed a Black module highly correlated with grain weight (Figure 6B,C), which was also enriched in starch synthesis and energy metabolism. This convergence of independent analytical approaches strengthened the credibility of identified candidates. By integrating Cluster 9, the Black module, and DEPs, we identified four key candidate genes (Figure 6D, Table 1), one of which was the previously characterized grain weight regulator *TaSBEIIa* [28]. Functional analysis using EMS-derived mutants further confirmed *TaYAK1-2D*, a dual-specificity protein kinase gene, as a positive regulator of grain weight (Figure 8C,I). These results not only validate the effectiveness of combining K-means and WGCNA for prioritizing causal genes from multi-omics data but also provide a set of promising targets for subsequent functional characterization and molecular breeding applications.

### 3.3. TaYAK1-2D Expands the Regulatory Landscape of Grain Weight

The identification of TaYAK1s as positive regulators introduces protein kinase-mediated signaling into the grain weight control network. While transcriptional and ubiquitin-mediated regulation have been extensively studied in cereal grain development [39], kinase-based signaling represents a relatively less explored mechanism. In *Arabidopsis thaliana*. AtYAK1 participates in ABA signaling by phosphorylating annexin proteins (ANN1/ANN2), thereby positively regulating drought stress responses and seedling growth [40,41]. In addition, within the TOR-YAK1 pathway, AtYAK1 inhibits cell cycle progression and promotes differentiation, thereby controlling organ size [42]. Our identification of *TaYAK1-2D* indicates that phosphorylation-mediated signaling directly modulates grain development. Although its precise phosphorylation targets remain unclear, *TaYAK1-2D* likely functions upstream of or parallel to known pathways, potentially phosphorylating transcription factors, metabolic enzymes, or cell cycle regulators to influence cell proliferation and expansion in developing grains. Further studies using gene-edited and overexpression materials will help clarify the mechanistic basis of *TaYAK1-2D*-mediated grain weight regulation and open new research avenues in signaling-mediated control of grain size and weight.

Furthermore, sequence alignment revealed that TaYAK1 on chromosome 2D exhibits the highest degree of divergence compared to its homologs on chromosomes 2A and 2B (Figure S4). This divergence suggests that *TaYAK1-2D* may have undergone functional specialization, potentially acquiring a distinct regulatory role in grain development. In contrast, TaYAK1-2A and TaYAK1-2B may either lack a direct function in regulating grain development or exert a substantially weaker effect compared to TaYAK1-2D.

### 3.4. Breeding Potential of TaYAK1-2D Variants

Marker-assisted selection (MAS) has become an efficient strategy for genetic improvement in crops, particularly for enhancing key agronomic traits such as grain weight [43] and grain filling [44]. The identification of *TaYAK1-2D* and its favorable haplotype *TaYAK1-2D-Hap2* provides a valuable genetic resource for wheat molecular breeding. To facilitate its utilization, we developed the functional marker *InDel125*, which reliably distinguishes *TaYAK1-2D*-*Hap2* from other haplotypes and enables efficient selection of high-grain-weight genotypes in breeding programs via MAS. Further enhancement of yield potential could be achieved by pyramiding *TaYAK1-2D-Hap2* with other known grain weight favorable alleles, such as *TaCAMTA2* [45], *TaSRK* [46], and *TaGDSL-7D* [47], or genes controlling grain dimensions, potentially resulting in synergistic improvements in grain yield. Moreover, the widespread distribution of *TaYAK1-2D-Hap2* across China’s major wheat-growing regions, along with its high frequency in both landraces and modern cultivars (Figure 9G,H), suggests it has undergone strong artificial selection during wheat domestication and improvement. As a robust and easy-to-score marker, *InDel125* can be readily integrated into existing MAS systems, providing breeders with an effective tool to accelerate the development of high-yielding wheat varieties.

## 4. Conclusions

This study established that early grain development, specifically at 7 DPA, serves as a decisive developmental window during which differential regulation of carbohydrate metabolism establishes the foundation for final grain weight in wheat. Through integrated multi-omics analysis, we identified *TaYAK1-2D* as a previously unrecognized positive regulator of grain weight and developed a functional marker, *InDel125*, for its superior haplotype, *TaYAK1-2D-Hap2*. Our study thus delivers not only a novel genetic determinant of grain weight but also a validated genetic marker that bridges the gap between gene discovery and breeding application.

## 5. Materials and Methods

### 5.1. Plant Materials and Field Trials

Jing 411 (J411), a leading wheat cultivar widely cultivated in the Northern China Plain since the 1990s, is characterized by its large grain size. KN9204, released in 2003, typically produces round but smaller grains. To develop NILs differing specifically in grain weight, KJ45, a large-grain recombinant inbred line (F8) derived from a KN9204 × J411 cross [48], was selected as the donor parent and backcrossed with KN9204. After two rounds of backcrossing followed by three generations of selfing, a pair of NILs with contrasting grain weight were obtained from the BC_3_F_3_ population: NIL1 (carrying large grain alleles) and NIL2 (carrying small grain alleles). These two NILs were used for all subsequent experiments.

Field trials were conducted at Zhaoxian Experimental Station, Shijiazhuang Academy of Agricultural and Forestry Sciences (37°75′ N, 114°78′ E). A randomized block design was carried out with three replications. Each plot contained three 2.0 m rows spaced 10 cm apart, with 20 seeds sown uniformly per row. All field management practices, including irrigation, followed standard procedures as previously described [49].

### 5.2. Phenotypic Evaluation

Agronomic traits of NIL1 and NIL2 were evaluated following the established method [49]. Measured parameters included PH, SPP, SL, SN, GNPS, TGW, GL, and GW. The same methods were applied to characterize EMS-induced mutants of KN9204. At the physiological maturity stage, a minimum of ten representative plants were selected from the central area of the middle row in each plot for agronomic traits assessment. Post-harvest analysis of TGW, GL, and GW was conducted using an SC-G multifunctional seed analyzer (Wanshen Detection, Hangzhou, China).

### 5.3. RNA-Seq and Transcriptomic Data Analysis

Transcriptomic profiling was performed using NIL1 and NIL2 plants. Seeds were collected from outer florets of the central spikelet on the main spike at four developmental stages: 7, 14, 21, and 28 DPA. For each time point and genotype, three biological replicates were obtained, with each replicate consisting of 20 seeds. All samples were snap-frozen in liquid nitrogen immediately after collection. Total RNA was isolated using TRIzol reagent according to the manufacturer’s instructions. In brief, tissue samples were pulverized in liquid nitrogen, then mixed with TRIzol reagent to lyse cells and release RNA, followed by chloroform addition to separate RNA from DNA and protein. RNA was precipitated with isopropanol, washed with ethanol, and resuspended in RNase-free water. RNA purity and integrity were verified before the samples were submitted to Shanghai Majorbio Bio-pharm Technology Co., Ltd. (Shanghai, China) for subsequent cDNA library construction and sequenced on the Illumina NovaSeq 6000 platform using 150 bp paired-end sequencing. Differential expression analysis was conducted using in-house established bioinformatics pipelines. In brief, raw RNA-seq reads were quality-controlled and adapter-trimmed using the fastp package to generate high-quality clean reads [50]. These reads were then mapped to the Chinese Spring reference genome V1.0 [51] using Hisat2. Gene expression levels were quantified in fragments per kilobase of transcript per million mapped reads (FPKM) and normalized by the trimmed mean of the M-values (TMM) method [52]. DEGs were identified with the DESeq2 package, applying a threshold of adjusted *p*-value (*padj*) < 0.05 and |log_2_FC| ≥ 1. Functional annotation of genes was performed using EggNOG-mapper, and enrichment analyses of GO terms and KEGG were conducted with the clusterProfiler R package v4.16.0. Co-expression networks were constructed using the WGCNA package in R following established methodologies [53].

### 5.4. TMT-Based Quantitative Proteomic Analysis

The proteomic analysis was performed using the same biological materials as the RNA-seq analysis, with three independent replicates per sample. Samples were processed by Shanghai Meiji Biomedical Technology Co., Ltd. (Shanghai, China) using the Tandem Mass Tag (TMT) isobaric labeling system (Thermo Fisher, Waltham, MA, USA) [54] for differential protein quantification. Following LC-MS/MS, the raw mass spectrometry data were processed for protein identification and quantification analysis following a standard procedure. Briefly, after enzymatic digestion, peptide samples were separated on a C18 column and analyzed by LC-MS/MS. The resulting spectra were processed using MaxQuant software v2.0 to identify and quantify proteins, generating protein expression profiles. Functional annotation was then performed through GO enrichment analysis using the DAVID database, while pathway analysis was conducted with the KEGG database. Significantly, DEPs were identified using technical replicates for each sample unit, and Student’s *t*-test was applied for parallel comparison. Proteins with *p*-value < 0.05 were considered significantly differentially expressed. The selected DEPs were subsequently subjected to GO and KEGG enrichment analysis [55] to elucidate their functional profiles and pathway associations.

### 5.5. RNA Extraction and RT-qPCR Validation

Total RNA was isolated using the RNAiso Plus Reagent (Takara Bio, Beijing, China) following the manufacturer’s instructions. cDNA was synthesized using the FastQuant RT Kit (Tiangen, Beijing, China), and quantitative PCR was performed on a LightCycler 96 system (Roche Applied Science, Penzberg, Germany) using SYBR Premix Ex Taq (Takara Bio, Beijing, China). The *TaActin* gene was used as the internal control, and relative expression levels were calculated using the 2^−ΔΔCT^ method [56]. Three biological replicates were used, and primer sequences are provided in Table S1.

### 5.6. Phylogenetic Analysis

Homologous protein sequences were retrieved from public databases, including Ensembl Plants and NCBI GenBank, across representative species: *Triticum aestivum*, *T. urartu*, *T. dicoccoides*, *Aegilops tauschii*, *Hordeum vulgare*, *Oryza sativa*, *Setaria italica*, *Zea mays*, and *Arabidopsis thaliana*. Multiple sequence alignment was conducted using ClustalW v2.1 [57], followed by trimming with trimAl v1.4 to retain core conserved regions. A maximum-likelihood phylogenetic tree was constructed in MEGA12 [58] and visualized using FigTree v1.4.4, where branches were scaled, nodes were annotated with bootstrap values, and labels were formatted. Final high-resolution vector graphics were exported for the final presentation.

### 5.7. Haplotype Association Analysis

Haplotype association analyses were performed using two independent wheat panels: the Chinese mini-core collection (MCC) [3] and a set of 132 wheat accessions from the Yellow and Huai River valley regions [59]. Phenotypic data for the MCC were collected from three environments: Luoyang (111°60′ E, 33°80′ N), Henan Province, China, in 2002 and 2005, and Shunyi (116°65′ E, 40°13′ N), Beijing, China, in 2010. For the 132 wheat accessions, phenotypes were evaluated across five environments: E1–E3 in Luancheng (114°41′ E, 37°53′ N) in 2015–2017, E4 in Anyang (113°37′ E, 35°12′ N) in 2017, and E5 in Beijing (116°65′ E, 40°13′ N) in 2017. Further details on population composition and field trials are provided in the cited references [3,59].

Haplotypes were discriminated using complementary PCR and KASP markers designed based on a 3′ UTR InDel (*InDel125*) and a closely linked SNP within *TaYAK1-2D*. For *InDel125* genotyping, PCR products were separated on 2% agarose gel at 100 V for 30–40 min, imaged under UV, and scored by fragment size: *TaYAK1-2D-Hap1* yields a 654 bp band, whereas *TaYAK1-2D-Hap2* produces a 529 bp band. KASP genotyping was performed following the standard fluorescent endpoint detection protocol.

The haplotype–trait association analysis was conducted as previously described [3]. Following genotyping, variance analyses were performed using SPSS 12.0 software (SPSS Inc., Armonk, NY, USA), with phenotypic traits compared among haplotypes via one-way ANOVA and Tukey’s test (*, *p* < 0.05; **, *p* < 0.01).

### 5.8. Statistical Analysis

Statistical analyses were performed on all previously described phenotypic data. Differences in TGW, GL, GW, PH, SPP, SL, SN, and GNPS were assessed by one-way ANOVA using Tukey’s test in SPSS Statistics 20.0 (IBM Corporation, Armonk, NY, USA).

## Figures and Tables

**Table 1:** Functional annotations of four candidate genes.

Gene ID	Gene Name	Location	Description
*TraesCS2A02G192900*	*TaERF1-2A*	chr2A:161270205-161274276(−)	Eukaryotic peptide chain release factor subunit 1-3 [UniProtKB/Swiss-Prot:P35614]
*TraesCS2D02G290800*	*TaSBEIIa-2D*	chr2D:372924177-372935106(−)	1,4-alpha-glucan-branching enzyme 1, amyloplastic [UniProtKB/Swiss-Prot:Q41058]
*TraesCS2D02G433100*	*TaYAK1-2D*	chr2D:544873315-544881090(+)	Dual specificity protein kinase YAK1 homolog [UniProtKB/Swiss-Prot:Q8RWH3]
*TraesCS5B02G400000*	*TaPho1-5B*	chr5B:577301899-577309490(−)	Alpha-glucan phosphorylase L isozyme, amyloplastic [UniProtKB/Swiss-Prot:P27598]

## Data Availability

The transcriptome data used in this study were deposited in the Genome Sequence Archive (https://bigd.big.ac.cn/gsa, (accessed on 11 October 2025)) under the accession number CRA031030. The proteome data were deposited in the Open Archive for Miscellaneous Data (https://ngdc.cncb.ac.cn/omix/, (accessed on 11 October 2025)) under the accession number OMIX012235. The data are contained within the article and the Supplementary Materials.

## Supplementary Materials

The following supporting information can be downloaded at: https://www.mdpi.com/article/10.3390/plants14243868/s1, **The Supplemental Tables include Tables S1–S12**. **Table S1.** Primers used in this study. **Table S2**. GO enrichment analysis of 18 clusters. **Table S3**. KEGG enrichment analysis of 18 clusters. **Table S4**. GO enrichment analysis of 12 modules. **Table S5**. KEGG enrichment analysis of 12 modules. **Table S6**. Mutation site information of *TaYAK1* mutants. **Table S7**. The 43 wheat accessions used for *TaYAK1-2D* genome sequence analysis in this study. **Table S8**. The populations used for association analysis and the distribution of alleles in the Chinese mini-core collection. **Table S9**. The populations used for the association analysis of 132 wheat accessions. **Table S10**. Marker/trait association analysis of *TaYAK1-2D* haplotypes in 157 landraces of MCC. **Table S11**. Marker/trait association analysis of *TaYAK1-2D* haplotypes in 86 modern cultivars of MCC. **Table S12**. Marker/trait association analysis of *TaYAK1-2D* haplotypes of 132 wheat accessions grown in five environments. **The Supplemental Figures include Figures S1–S6**. **Figure S1**. Expression and functional analysis of carbon metabolism-related genes during early grain development. **Figure S2**. Correlation heatmap analysis of gene expression during grain development. **Figure S3**. Expression profiling of grain weight-related candidate genes involved in the carbon metabolic pathway. **Figure S4**. Multiple sequence alignment of TaYAK1 homologs across monocot species. **Figure S5**. Identification of homozygous mutations in *TaYAK1* genes. **Figure S6**. Haplotype typing results of the natural population of TaYAK1-2D.
