# Supplementary material for: Integrated Multi-Omics Reveals the Molecular Basis Underlying Wheat Grain Development and Identifies TaYAK1-2D as a Positive Grain Weight Regulator
Source: Plants (Basel). 2025 Dec 18;14(24):3868. doi: 10.3390/plants14243868 (PMC12736556; doi:10.3390/plants14243868)
Supplement: Supplementary file 1 [file plants-14-03868-s001.zip › Supplemental FiguresRE.pdf]

Supplementary data:

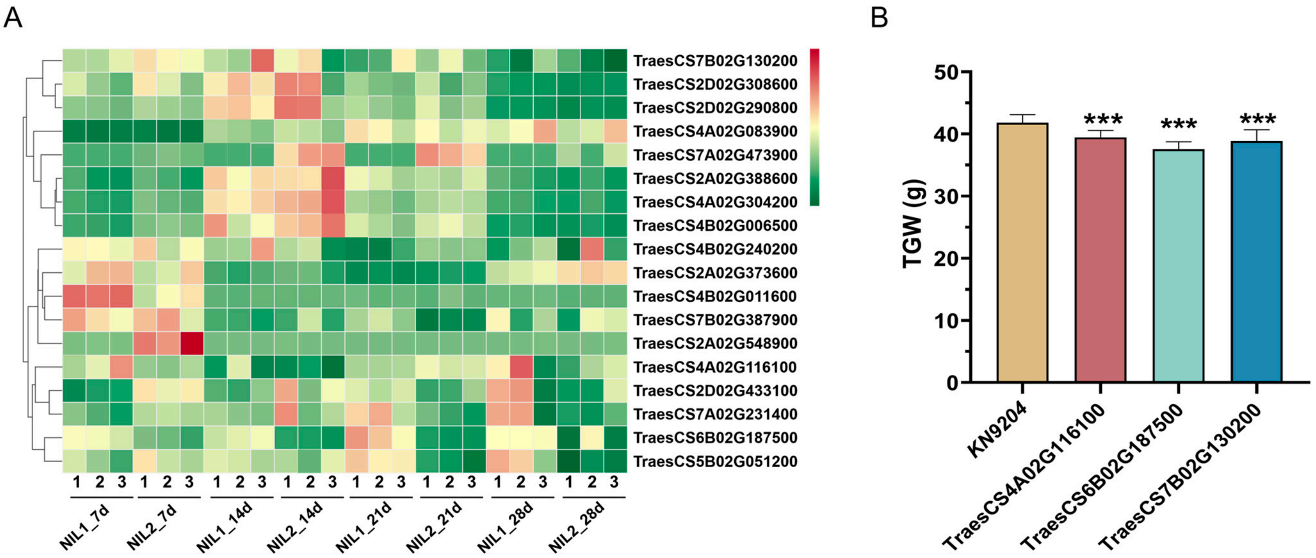

Figure S1. Expression and functional analysis of carbon metabolic-related genes during early grain development. (**A**) Expression patterns of differentially expressed genes involved in carbon metabolic pathways at 7 DPA in NIL1 and NIL2. (**B**) Comparative analysis of TGW between wild-type (KN9204) and EMS mutants of three carbon metabolic pathway-related genes. Data represent mean ± SD; *** $P < 0.001$ (versus wild-type, one-way ANOVA).

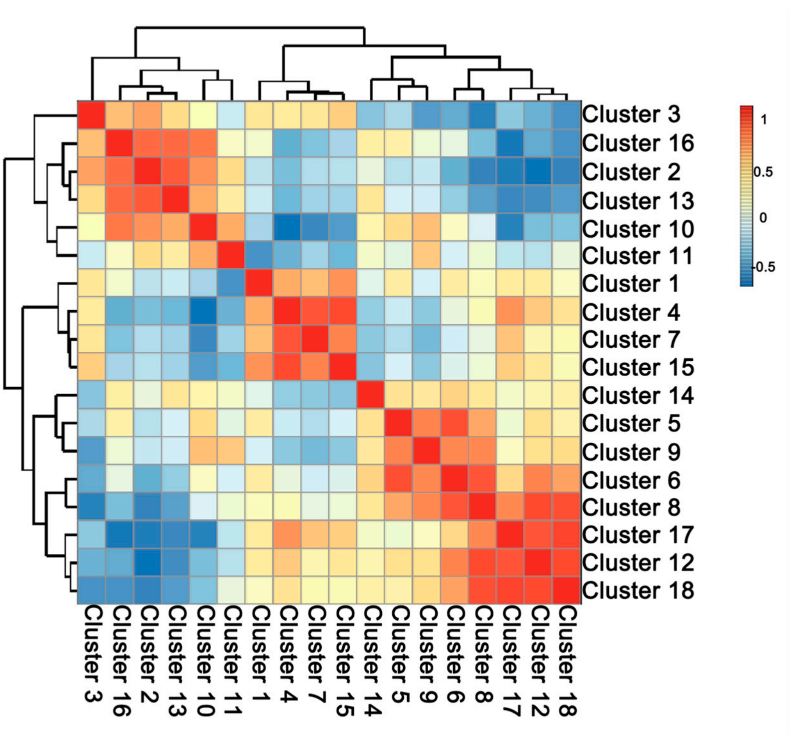

Figure S2. Correlation heatmap analysis of gene expression during grain development.

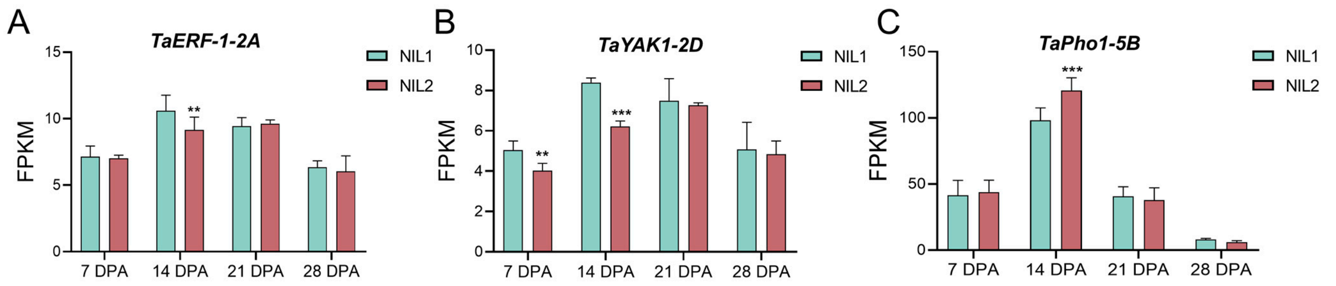

Figure S3. Expression profiling of grain weight-related candidate genes involved in carbon metabolic pathway. (**A**–**C**) Relative expression level of *TraesCS2A02G192900* (*TaERF-1-2A*), *TraesCS2D02G433100* (*TaYAK1-2D*), and *TraesCS5B02G400000* (*TaPho1-5B*) in developing grains of NIL1 and NIL2 at 7, 14, 21, and 28 DPA. Data represent as mean ± SD from three biological replicates; statistically significant differences are indicated by asterisks (** $P < 0.01$, *** $P < 0.001$, one-way ANOVA).

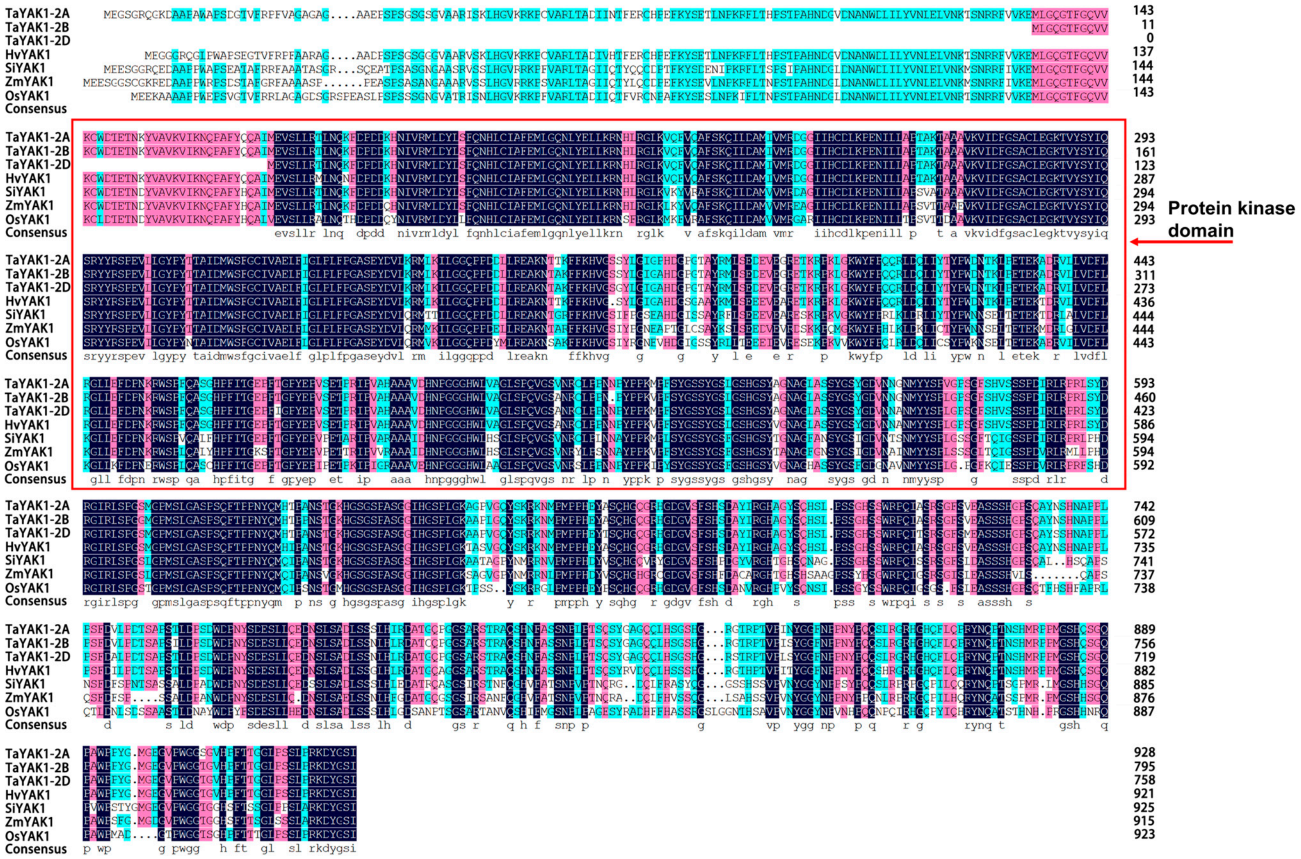

Figure S4. Multiple sequence alignment of TaYAK1 homologs across monocot species. The conserved protein kinase domain is highlighted with a red box. Species abbreviations: Ta: *Triticum aestivum*; Hv: *Hordeum vulgare*; Os: *Oryza sativa Japonica Group*; Zm: *Zea mays*; Bd: *Brachypodium distachyon*; Si: *Setaria italica*.

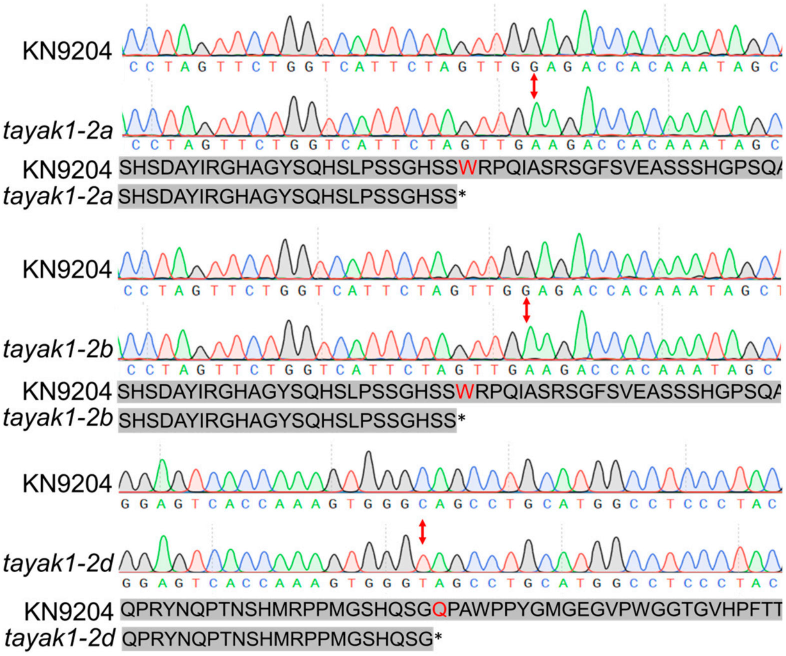

Figure S5. Identification of homozygous mutations in *TaYAK1* genes. Sequencing chromatograms and corresponding sequences from wild type (KN9204), and representative editing mutant lines. Red arrows indicate the positions of the mutated bases in each mutant.

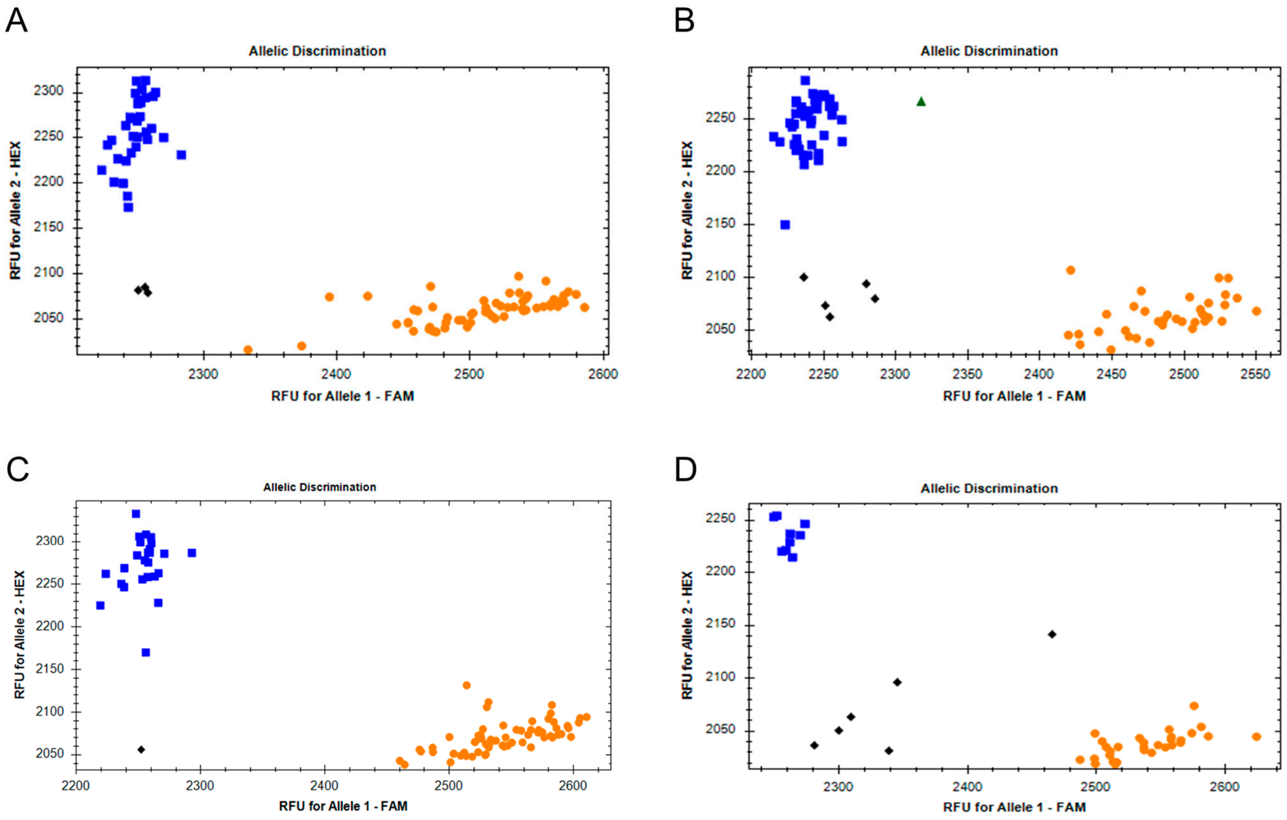

Figure S6. Haplotype typing results of natural population of TaYAK1-2D. (A–B) Marker analysis of *TaYAK1-2D* haplotypes in MCC. (C–D) Marker analysis of *TaYAK1-2D* haplotypes in 132 wheat accessions.
